# The Impact of Spectral and Temporal Degradation on Vocoded Speech Recognition in Early-Blind Individuals

**DOI:** 10.1523/ENEURO.0528-23.2024

**Published:** 2024-05-24

**Authors:** Hyo Jung Choi, Jeong-Sug Kyong, Jae Hee Lee, Seung Ho Han, Hyun Joon Shim

**Affiliations:** ^1^Department of Otorhinolaryngology-Head and Neck Surgery, Nowon Eulji Medical Center, Eulji University School of Medicine, Seoul 01830, Republic of Korea; ^2^Eulji Tinnitus and Hearing Research Institute, Nowon Eulji Medical Center, Seoul 01830, Republic of Korea; ^3^Sensory Organ Institute, Medical Research Institute, Seoul National University, Seoul 03080, Republic of Korea; ^4^Department of Radiology, Konkuk University Medical Center, Seoul 05030, Republic of Korea; ^5^Department of Audiology and Speech-Language Pathology, Hallym University of Graduate Studies, Seoul 06197, Republic of Korea; ^6^Department of Physiology and Biophysics, School of Medicine, Eulji University, Daejeon 34824, Republic of Korea

**Keywords:** electroencephalogram, spectral degradation, speech recognition, temporal degradation, visual deprivation, vocoder

## Abstract

This study compared the impact of spectral and temporal degradation on vocoded speech recognition between early-blind and sighted subjects. The participants included 25 early-blind subjects (30.32 ± 4.88 years; male:female, 14:11) and 25 age- and sex-matched sighted subjects. Tests included monosyllable recognition in noise at various signal-to-noise ratios (−18 to −4 dB), matrix sentence-in-noise recognition, and vocoded speech recognition with different numbers of channels (4, 8, 16, and 32) and temporal envelope cutoff frequencies (50 vs 500 Hz). Cortical-evoked potentials (N2 and P3b) were measured in response to spectrally and temporally degraded stimuli. The early-blind subjects displayed superior monosyllable and sentence recognition than sighted subjects (all *p* < 0.01). In the vocoded speech recognition test, a three-way repeated-measure analysis of variance (two groups × four channels × two cutoff frequencies) revealed significant main effects of group, channel, and cutoff frequency (all *p* < 0.001). Early-blind subjects showed increased sensitivity to spectral degradation for speech recognition, evident in the significant interaction between group and channel (*p* = 0.007). N2 responses in early-blind subjects exhibited shorter latency and greater amplitude in the 8-channel (*p* = 0.022 and 0.034, respectively) and shorter latency in the 16-channel (*p* = 0.049) compared with sighted subjects. In conclusion, early-blind subjects demonstrated speech recognition advantages over sighted subjects, even in the presence of spectral and temporal degradation. Spectral degradation had a greater impact on speech recognition in early-blind subjects, while the effect of temporal degradation was similar in both groups.

## Significance Statement

Like sighted people, blind individuals can experience hearing impairment as they age. Therefore, studying speech recognition in the context of degraded spectral/temporal resolution is crucial for simulating individuals with both hearing and visual impairments. The current study is the first to compare speech recognition and relevant cortical-evoked potentials between early-blind subjects and age- and sex-matched sighted subjects under conditions of degraded auditory spectral and temporal resolution. The results have implications for designing interventions and support systems for individuals with combined visual and hearing impairments.

## Introduction

Early-blind individuals have an increased prevalence of absolute pitch (Hamilton et al., 2004) and better abilities in performing pure-tone pitch discrimination (Gougoux et al., 2004; Wan et al., 2010; Voss and Zatorre, 2012), spectral ripple discrimination (Shim et al., 2019), music and speech pitch discrimination (Arnaud et al., 2018), and pitch–timbre categorization (Wan et al., 2010), when compared with sighted individuals. Early-blind individuals also exhibit better temporal-order judgment ability (Weaver and Stevens, 2006), temporal auditory resolution ability using gap detection (Muchnik et al., 1991), temporal modulation detection (Shim et al., 2019), and temporal attention for stimulus selection (Röder et al., 2007). Some studies found no difference in the gap detection threshold (Weaver and Stevens, 2006; Boas et al., 2011) and temporal bisection (Vercillo et al., 2016; Campus et al., 2019; Gori et al., 2020) between blind and sighted individuals. However, prior studies comparing speech recognition in early-blind and sighted individuals have yielded inconclusive results (Gougoux et al., 2009; Ménard et al., 2009; Hertrich et al., 2013; Arnaud et al., 2018; Shim et al., 2019).

Blind individuals rely heavily on their hearing to communicate, navigate, and access information without visual cues. Therefore, in environments where sound information is distorted, blind individuals face much more severe challenges compared with those who are not visually impaired. In our previous study (Bae et al., 2022), it was clear that there were significant differences in speech perception between sighted individuals under audio–visual (AV) condition and blind individuals under auditory-only (AO) conditions. However, under the same AO conditions, blind individuals demonstrated comparable performance to sighted individuals and even showed a superior trend under low signal-to-noise ratios (SNRs; high noise levels). Our first hypothesis was that as SNR decreases, the speech recognition ability of early-blind individuals would exhibit even greater superiority over sighted individuals.

Spectral and temporal degradation in sound can pose challenges to normal sound perception and comprehension. Distorted sound makes it difficult for accurate sound information coding throughout the entire auditory system, from cochlear hair cells to auditory brain neurons. However, no studies have yet compared speech recognition between blind and sighted individuals under conditions of degraded auditory spectral and temporal resolution. Given that early-blind individuals exhibit superior spectral and temporal resolution compared with sighted individuals (Shim et al., 2019), we hypothesized that blind individuals would still exhibit superior speech recognition compared with sighted individuals under conditions of degraded auditory spectral and temporal resolution in AO situations.

To verify these hypotheses, we examined whether speech recognition of monosyllabic words and sentences differs between early-blind and sighted individuals in the case of decreasing SNR. Furthermore, we compared vocoded speech recognition between early-blind and sighted individuals. The noise vocoder utilized 4, 8, 16, and 32 channels to simulate spectral degradation and set cutoff frequencies at 50 and 500 to simulate temporal degradation.

Finally, we used the “semantic oddball paradigm” to investigate the N2 and P3b responses in the cortical-evoked potentials. N2 is a negative-going wave that starts ∼200–300 ms poststimulus (Folstein and Van Petten, 2008) and is a sensitive index for examining the course of semantic and phonological encoding during implicit picture naming with the go/no-go paradigm (Schmitt et al., 2000) or listening to sound with the oddball paradigm (Finke et al., 2016; Voola et al., 2023). P3b, which occurs between 250 and 800 ms, exhibits a variable peak dependent on the individual response, and greater amplitudes are typically observed over the parietal brain regions on the scalp (Polich, 2007; Levi-Aharoni et al., 2020). P3b is associated with updating working memory, and prolonged latencies may represent slower stimulus evaluation (Beynon et al., 2005; Henkin et al., 2015). With these experiments, we sought to compare the impact of spectral and temporal degradation on vocoded speech recognition and the cortical auditory responses between early-blind individuals and sighted individuals. In our previous study, we confirmed that the N2 and P3b responses reflect the channel effect in the cortex using a one-syllable oddball paradigm with animal and nonanimal stimuli across four vocoder conditions (4, 8, 16, or 32 channel bands), indicating less efficient semantic integration due to reduced spectral information in speech (Choi et al., 2024). Therefore, in this study, we compared the N2 and P3b responses between early-blind and sighted individuals using the same vocoded speech recognition paradigm with four different numbers of channels and two temporal envelope cutoff frequencies, enabling us to assess semantic processing.

## Materials and Methods

### Subjects

The study population included a group of 25 early-blind subjects (30.19 ± 4.83 years; male:female ratio, 14:11) and a control group of 25 age- and sex-matched sighted subjects (30.00 ± 6.58 years; male:female ratio, 14:11). All the subjects in both groups were right-handed, aged <40 years, had normal hearing thresholds in both ears (≤20 dB hearing level at 0.25, 0.5, 1, 2, 3, 4, and 8 kHz), and had no neurological or ontological problems. In the early-blind group, only those who were blind at birth or who became blind within 1 year of birth and those classified in Categories 4 and 5 according to the 2006 World Health Organization guidelines for the clinical diagnosis of visual impairment (Category 4, “light perception” but no perception of “hand motion”; Category 5, “no light perception”) were included (World Health Organization, 2006). Table 1 provides the characteristics of the blind subjects.

**Table 1.** Clinical characteristics for the early-blind subjects

Subject	Age (years)	Onset	Sex	Visual acuity	Cause of blindness
B01	32	Birth	F	Light perception	Retinopathy of prematurity
B02	25	Birth	M	No light perception	Optic atrophy
B03	28	Birth	F	Light perception	Optic atrophy
B04	22	Birth	F	No light perception	Retinopathy of prematurity
B05	27	Birth	M	Light perception	Microphthalmos
B06	39	Birth	M	Light perception	Optic atrophy
B07	32	Birth	M	No light perception	Persistent hyperplastic primary vitreous
B08	22	Birth	F	No light perception	Retinopathy of prematurity
B09	28	Birth	M	No light perception	Retinopathy of prematurity
B10	39	Birth	F	No light perception	Cataract
B11	34	Birth	F	Light perception	Retinoblastoma
B12	28	Birth	M	No light perception	Retinopathy of prematurity
B13	29	Birth	F	No light perception	Retinopathy of prematurity
B14	26	Birth	M	No light perception	Corneal opacity
B15	30	Birth	F	No light perception	Retinopathy of prematurity
B16	34	Birth	M	Light perception	Retinopathy of prematurity
B17	35	Birth	M	Light perception	Cause unknown
B18	31	Birth	M	Light perception	Cause unknown
B19	36	Birth	F	Light perception	Retinopathy of prematurity
B20	34	Birth	M	NO light perception	Meningitis
B21	37	Birth	F	NO light perception	Optic atrophy
B22	25	Birth	M	NO light perception	Cause unknown
B23	27	Birth	M	NO light perception	Retinopathy of prematurity
B24	32	Birth	M	Light perception	Cause unknown
B25	26	Birth	M	Light perception	Xanthochromism

The study was conducted in accordance with the Declaration of Helsinki and the recommendations of the Institutional Review Board of Nowon Eulji Medical Center, with written informed consent from all subjects. Informed consent was obtained verbally from the blind subjects in the presence of a guardian or third party. The subjects then signed the consent form, and a copy was given to them.

### Behavioral tests

The early-blind and sighted subjects performed four behavioral tests: digit span test (Wechsler, 1987), monosyllable recognition in noise (Bae et al., 2022), Korean Matrix sentence recognition in noise (Kim and Lee, 2018; Jung et al., 2022), and vocoded speech recognition (Choi et al., 2024). All tests were conducted in a soundproof room with an audiometer (Madsen Astera 2; GN Otometrics) and a loudspeaker installed in the frontal direction at 1 m from the subject's ear.

#### Digit span test

The digit span test was conducted to examine the effect of working memory on central auditory processing. All digit span tests consisted of the digits 1–9, and the digit sets were presented consecutively with an increasing number of digits from 3 to 10. The digit sets were randomly generated, and the same number of digits was repeated twice. The threshold of the digit span test was determined to be at least two incorrect responses to the previous digit series. The set of digits was presented at 70 dB sound pressure level (SPL), with a 1 s interval between sets. The subjects were asked to repeat the set of digits forward and backward.

#### Speech recognition in noise

The monosyllabic word recognition in noise test was performed at five SNRs (−18, −16, −12, −8, and −4) using five lists, each containing 25 Korean monosyllabic words, which were spoken by a male speaker, and eight-talker babble noise. In our previous study (Bae et al., 2022), we compared the speech perception of early-blind and sighted subjects across five different SNRs (−18, −16, −12, −8, and −4) using the same monosyllable set as in the current research. Monosyllable perception in noise tended to be better in early-blind subjects than in sighted subjects at SNR of −8; however, the results at SNR −4, 0, +4, and +8 did not differ. Therefore, in this study, we designed conditions with relatively lower SNRs (higher noise levels).

The mixture of the target word and the noise stimuli was delivered by a loudspeaker located 1 m in front of the subjects, and the subjects were asked to repeat the words while ignoring the noise. The noise level was fixed at 70 dB SPL, and the level of the target monosyllable words was varied. The word-in-noise recognition scores were calculated as the percentage of correctly repeated words in each SNR condition.

To measure sentence-in-noise recognition, we used the Korean Matrix sentence recognition test (Kim and Lee, 2018; Jung et al., 2021, 2022). All the Korean Matrix sentences used are semantically unpredictable, but they have the same grammatical structure (name, adjective, object, numeral, and verb) because each sentence was generated using a 5 × 10 base word matrix (10 names, 10 adjectives, 10 nouns, 10 numerals, and 10 verbs). The general principles and applications of the Korean Matrix sentence-in-noise recognition tests are described in previous studies (Wagener and Brand, 2005; Akeroyd et al., 2015; Kollmeier et al., 2015). We utilized two types of noise in the Korean Matrix sentence-in-noise test: speech-shaped noise (SSN) and the International Speech Test Signal (ISTS). The SSN noise was generated by superimposing the Korean Matrix sentences, so the long-term spectrum of speech and SSN was the same. The ISTS noise (Holube et al., 2010) is considered nonintelligible speech noise because it consists of randomly remixed speech segments (100–600 ms) from six languages, which are spoken by six different female talkers reading *The North Wind and the Sun*.

The Korean Matrix sentence recognition test was conducted using the Oldenburg Measurement Applications software (HörTechg). The test sentences and noise were presented through a Fireface UCX digital-to-analog converter (RME Audio Interfaces), and the stimuli were delivered by a loudspeaker located 1 m in front of the subjects. During the test, the noise level was fixed at 65 dB SPL, while the sentence level was adjusted according to the subject's response based on the maximum likelihood estimator (Brand and Kollmeier, 2002). Consequently, we measured the speech reception thresholds of 50% intelligibility by measuring the SNRs required to achieve 50% recognition.

#### Vocoded speech recognition

**Figure 1.**
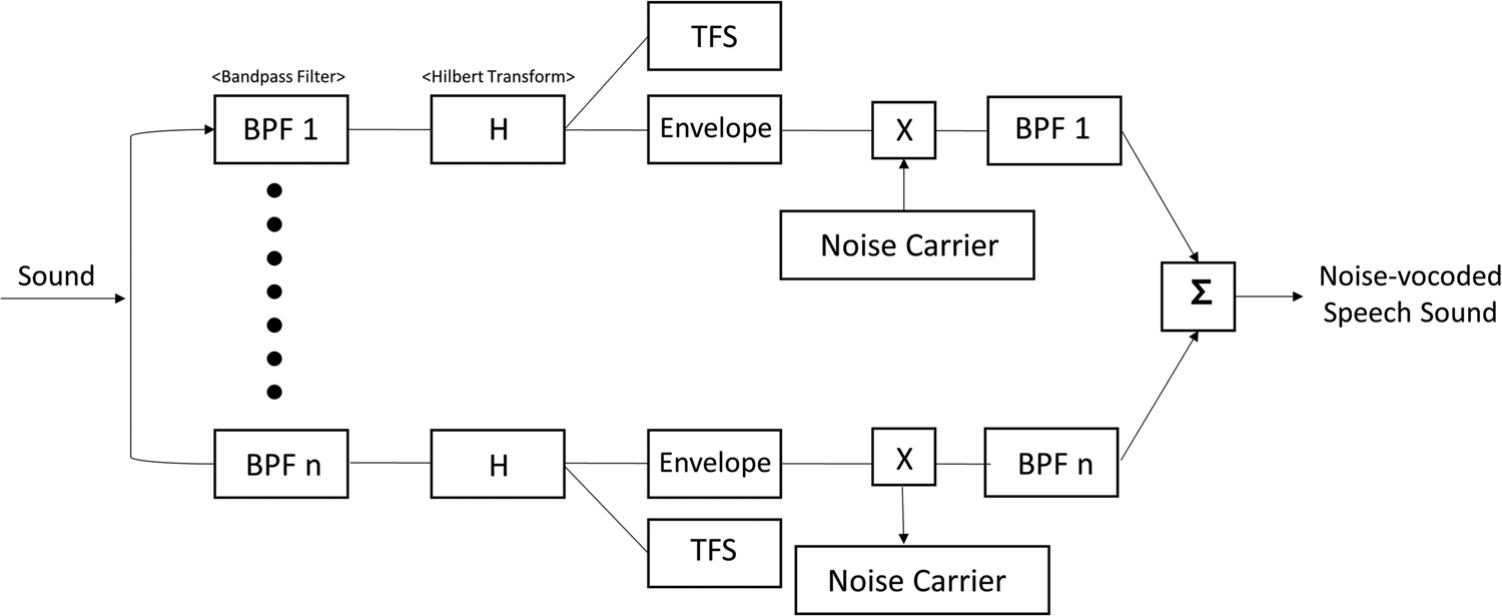
An illustration depicting the generation of the noise-vocoded signal. The input signals were bandpass filtered into 4 (BPF1), 8 (BPF2), 16 (BPH3), and 32 (BPF4) channel bands prior to Hilbert transformation. After separating the envelopes from the temporal fine structures, the vocoder speech signal was generated by adding a noise carrier to the envelopes.

**Figure 2.**
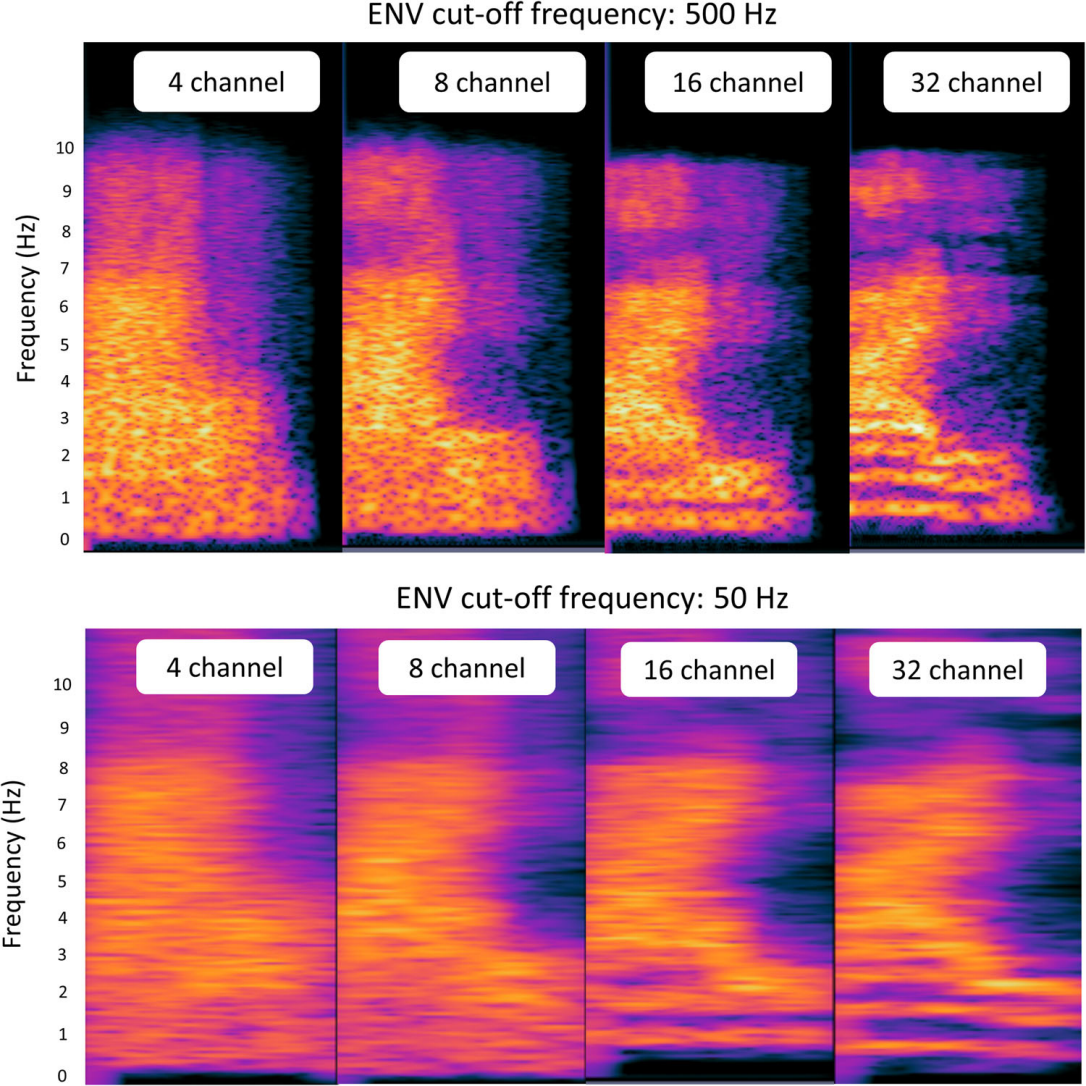
Spectrograms for the number of channels (4, 8, 16, and 32) at cutoff frequencies of 50 or 500 Hz. With fewer channel bands and a lower cutoff frequency, the speech becomes more spectrally degraded and difficult to understand.

Stimuli were recorded by a male speaker reading five lists of 25 monosyllabic Korean words in a soundproof booth using a lapel microphone (BY-WMA4 PRO K3, BOYA). All the recorded stimuli were sampled at a rate of 44,100 Hz, and the overall root mean square amplitude was set at −22 dB. Noise vocoding involves passing a speech signal through a filter bank to extract time-varying envelopes associated with the energy in each spectral channel. The extracted envelopes were then multiplied by white noise and combined after refiltering (Shannon et al., 1995). Figure 1 illustrates the method used to produce noise vocoding. Initially, the incoming signals were processed through bandpass filtering, generating multiple numbers of channel bands (4, 8, 16, or 32 channels). The cutoff frequencies of each bandpass filter were determined using a logarithmically spaced frequency range based on the Greenwood function (e.g., 80, 424, 1,250, 3,234, and 8,000 Hz for the four-channel test). The cutoff frequency of the low-pass filter for temporal envelope extraction was applied at both 50 and 500 Hz, depending on whether fundamental frequency (F0)-related periodicity cues were included (i.e., the absence of F0 cue at 50 Hz vs the presence of F0 cue at 500 Hz cutoff frequency). The central frequency of each channel was calculated as the geometric mean between the two corresponding cutoff frequencies associated with that specific channel. The collective input frequency ranged from 80 to 8,000 Hz. Subsequently, the amplitude envelope for each frequency band was extracted through half-wave rectification. Finally, we summed the data to generate the noise-vocoded session (Shannon et al., 1995; Faulkner et al., 2012; Evans et al., 2014). Vocoding was performed using a custom MATLAB script (2020a, MathWorks), in which the spectral detail decreased as the number of channel bands decreased, as shown in Figure 2. The target word was presented at 70 dB SPL by a loudspeaker located 1 m in front of the subjects, and the word recognition scores were calculated as the percentage of correctly repeated words.

### Electroencephalogram (EEG)

#### N2 and P3b

According to the semantic oddball paradigm, animal stimuli or nonanimal but sensible stimuli were delivered to the subjects. Overall, 70% of the trials were animal words (e.g., mouse, snake, and bear; all monosyllable in Korean). The remaining 30% consisted of monosyllable nonanimal words that are like animal words but belong to a different semantic category. The subjects sat comfortably in a soundproof booth and listened to the animal or nonanimal words in a random order. The researchers explained the subjects should press the button quickly and accurately when they hear a nonanimal word. By pressing a button on a nonanimal stimulus, the subjects were able to focus on the task. In each channel condition (4, 8, 16, and 32 channels), 210 animal words and 90 nonanimal words were implemented in six blocks, and the subjects listened to a total of 1,200 trials. The interstimulus interval was fixed at 2,000 ms, and a jitter of 2–5 ms was allowed. The order of presentation was randomized within the blocks, and the order of blocks was counterbalanced among subjects using the E-Prime software (version 3, Psychology Software Tools). Each subject had a 5 min break after completing each block. The subjects had a practice session, prior to starting the trials, to ensure they understood the task and ensure their muscles were relaxed. The intensity of the sound was fixed at 70 dB SPL when calibrated at the listener's head position, 1 m from the loudspeaker.

#### Procedure

Neural response was recorded across 31 AG-Ag/Cl sintered electrodes placed according to the international 10–20 system (Klem, 1999) and referenced FCz in an elastic 32-channel cap using the actiCHamp Brain Products recording system (BrainVision Recorder Professional, V.1.23.0001, Brain Products) in a dimly-lit, sound–attenuated, electrically shielded chamber. Electrooculogram and electrocardiogram were tagged to trace the subject's eye movement and heartbeat. EEG data were digitized online at a sampling rate of 1,000 Hz. All 32 electrodes were referenced to the algebraic average of all electrodes/channels and were therefore unbiased to any electrode position. The ground electrode was placed between electrodes Fp1 and Fp2. Software filters were set at low (0.5 Hz) and high (70 Hz) cutoffs. A notch filter at 60 Hz was set to prevent powerline noise, and the impedances of all scalp electrodes were kept below 5 kΩ using EEG electrode gel throughout the recording, following the manufacturer's instructions.

#### Data processing

**Figure 3.**
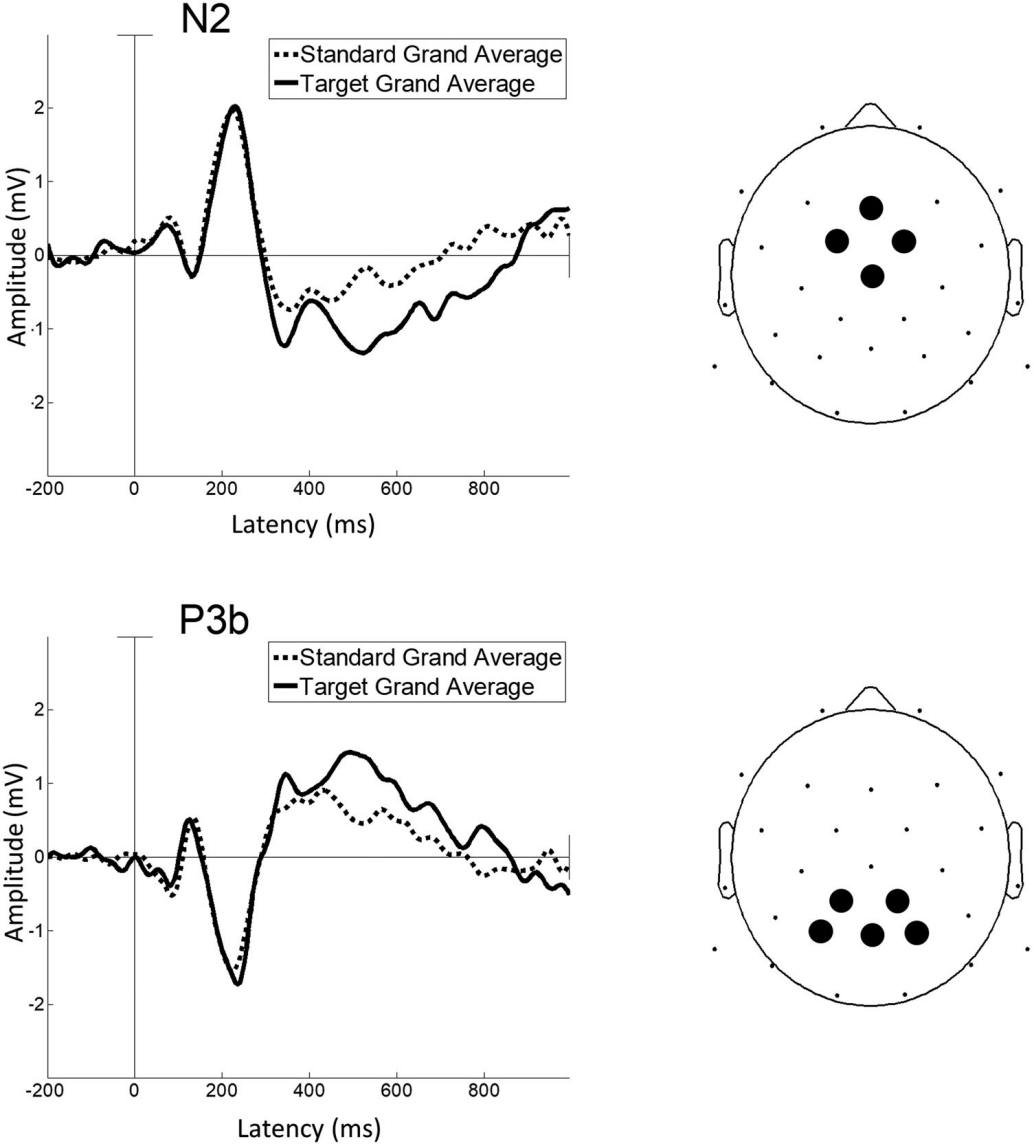
Sample waveforms averaged from the corresponding electrodes for N2 and P3b. For N2, it represents the average values obtained from Fz, FC1, FC2, and Cz channels. For P3b, it represents the average values obtained from CP1, CP2, P3, Pz, and P4 channels.

The data were preprocessed and analyzed with BrainVision Analyzer (version 2.0, Brain Products) and MATLAB R2019b (MathWorks) using EEGLAB v2021 (Delorme and Makeig, 2004) and FieldTrip (Oostenveld et al., 2011) toolboxes. EEG was filtered with a high-pass filter at 0.1 Hz (Butterworth filter with a 12 dB/oct roll-off) and a low-pass filter at 50 Hz (Butterworth filter with a 24 dB/oct roll-off). The first three trials were excluded from the analyses. Data were resampled at 256 Hz. Independent component analysis was used to reject artifacts associated with eyeblinks and body movement (average of four independent components; range, 3–6) and reconstructed (Makeig et al., 1997), transforming to the average reference. The EEG waveforms were time-locked to each stimulus onset and segmented from 200 ms prior to the stimulus onset to 1,000 ms after the stimulus onset. Baseline correction was also performed. Prior to averaging, bad channels were interpolated using a spherical spline function (Perrin et al., 1989), and segments with values ±70 µV at any electrode were rejected. All the subjects had data for at least 180–200 out of 210 usable standard trials and 78–86 usable target trials per vocoder channel. Based on the averaged waveform of the electrodes in the corresponding area in Figure 3, the N2 component was defined as the period 280–870 ms poststimulus onset, and the P3b component was defined as the period 280–840 ms poststimulus onset. An average wave file was generated for each subject for each condition. Based on the grand average computed across all conditions and participants, latency ranges for N2 and P3b were determined according to the literature, and the peak latency was measured using a half-area quantification, which may be less affected by latency jitter (Luck, 2014; Finke et al., 2016). Difference waveforms were constructed based on the subtraction of target stimuli from standard stimuli within conditions (Deacon et al., 1991). The area latency and amplitude of the N2 and P3b difference waveforms at each condition were compared. The time windows for N2 and P3b analysis were defined from each average waveform. In our data, the time windows for N2 and P3b were set as 280–870 ms and 280–840 ms, respectively. N2 was measured by averaging the signals from the frontocentral electrodes (Fz, FC1, FC2, and Cz), while P3b was measured using the parietal electrodes (CP1, CP2, P3, P4, and Pz), as outlined in Finke et al. (2016).

### Statistical analysis

We used the Mann–Whitney test to compare the differences in the digit span test between the early-blind and sighted subjects because the data did not follow a normal distribution based on the Kolmogorov–Smirnov test. Two-way repeated–measure analysis of variance (RM-ANOVA) was used to analyze the effects of group and SNRs on monosyllable recognition, as well as on the N2 and P3b components. The same method was used to examine the effects of group and type of noise on sentence recognition. We also used three-way RM-ANOVA to investigate the effect of group, number of channels, and envelope cutoff frequency. All statistical analyses were performed using the IBM SPSS software (ver. 25.0; IBM).

## Results

### Behavioral tests

#### Digit span test

**Figure 4.**
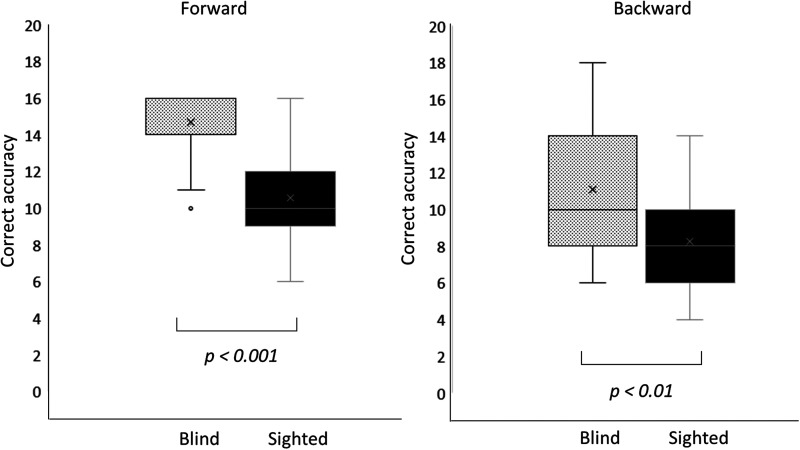
Digit span test. The correct score was higher in the early-blind group than in the sighted group in the forward (*p* < 0.001) and backward (*p* = 0.004) conditions.

The digit span test measures attention and working memory through forward and backward recall of digit sequences (Banken, 1985; Choi et al., 2014). In the forward test, early-blind subjects exhibited an average score of 14.7 ± 1.73 points, whereas sighted subjects scored an average of 10.6 ± 1.9 points. There was a statistically significant difference in the test accuracy between the two groups (*z* = −5.091; *p* < 0.001; Mann–Whitney test). The backward test revealed a score of 11.1  3.44 for early-blind participants and 8.3 ± 2.8 for their sighted subjects. There was a statistically significant difference in accuracy between the groups (*z* = −2.862; *p* = 0.004; Mann-Whitney test; Fig. 4). Notably, the early-blind subjects exhibited superior working memory to the sighted subjects.

#### Monosyllabic word-in-noise and sentence-in-noise recognition

**Figure 5.**
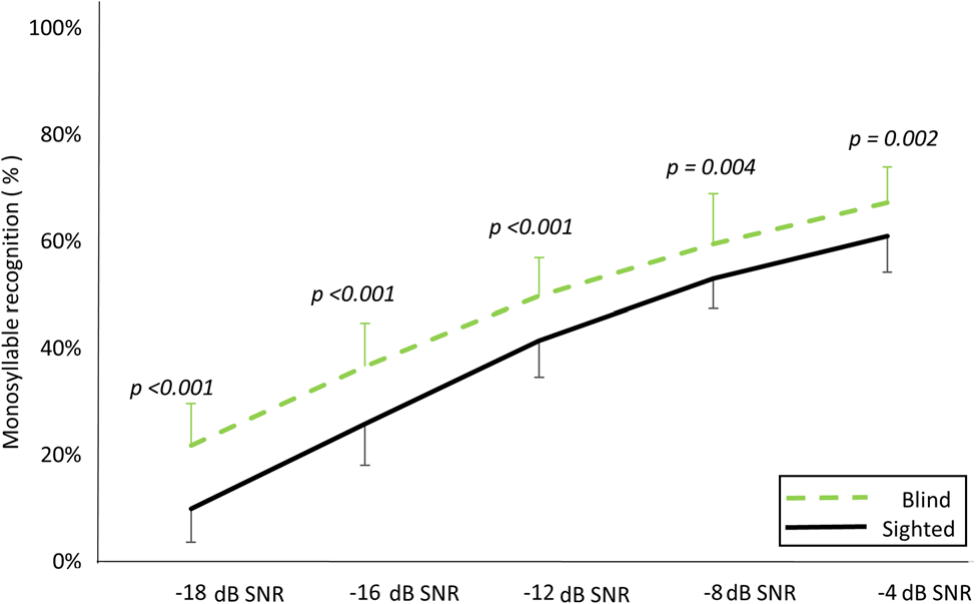
Monosyllable word recognition in noise. The early-blind subjects demonstrated better recognition compared with the sighted subjects in all SNR conditions (all *p* < 0.01).

To minimize redundant cues in speech recognition, we employed monosyllabic word recognition. Additionally, sentence-in-noise recognition was measured to reflect real-life conversational scenarios. The mixed two-way RM-ANOVA (two groups × five SNRs) for word-in-noise showed a significant main effect of group with the blind group performing better (*F*_(1, 48)_ = 46.511; *p* < 0.001) and for SNR (*F*_(4, 192)_ = 456.520; *p* < 0.001), without significant interaction between the two variables (*F*_(4, 192)_ = 1.927; *p* = 0.108; Table 2). In all SNRs, early-blind subjects showed superior word recognition compared with sighted subjects (−18 SNR, *p* < 0.001; −16 SNR, *p* < 0.001; −12 SNR, *p* < 0.001; −8 SNR, *p* = 0.004; −4 SNR, *p* = 0.002; Bonferroni-corrected *p* < 0.05; Fig. 5).

**Table 2.** ANOVA table of monosyllable word in noise

	Sum of square	df	Mean square	*F*	*p*	$\eta_{G}^{2}$
SNR	4,695.30	4.00	1,173.83	456.52	<0.001	0.905
Group	300.304	1	300.304	46.511	<0.001	0.492
SNR*Group	19.816	4	4.954	1.927	0.108	0.39
Residual	493.68	192.00	2.57			

**Figure 6.**
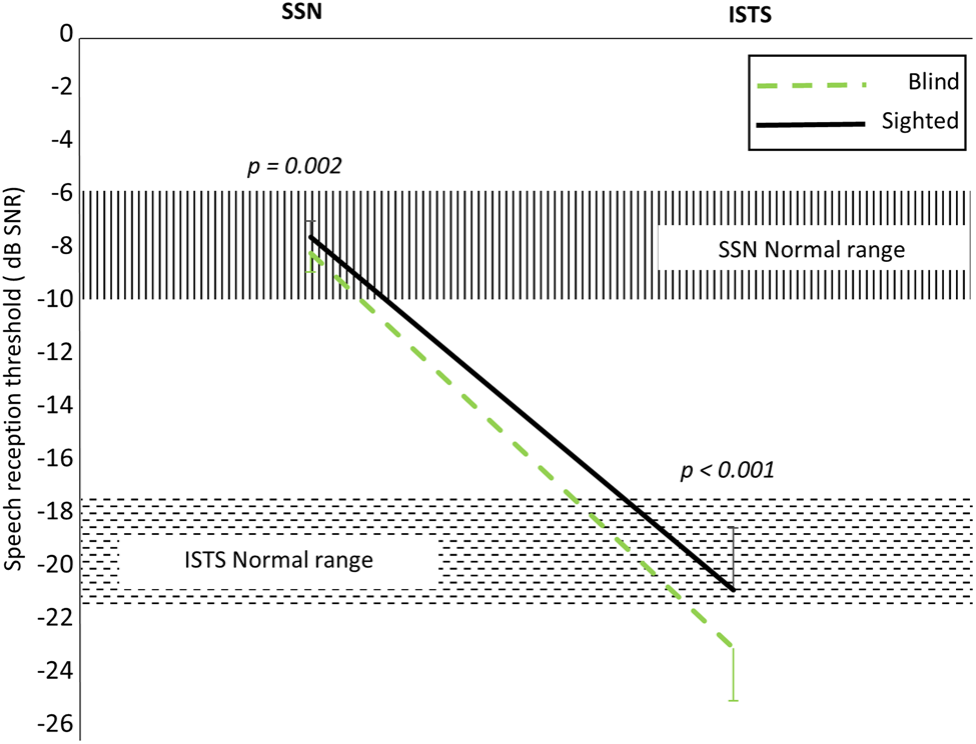
Sentence recognition in noise. The speech recognition threshold was significantly lower in the early-blind group compared with the sighted group for the SSN test (*p* = 0.002) and the ISTS noise test (*p* < 0.001).

The mixed two-way RM-ANOVA (two groups × two types of noise) for sentence recognition showed a significant main effect of group with the blind group performing better (*F*_(1, 48)_ = 16.627; *p* < 0.001) and noise type (*F*_(1, 48)_ = 2,298.198; *p* < 0.001), and there was a significant interaction between the two factors (*F*_(1, 48)_ = 7.349; *p* = 0.009; Table 2). In the post hoc tests, the early-blind group showed better recognition than the sighted group for both SSN and ISTS (*p* = 0.002 and *p* < 0.001, respectively; Bonferroni-corrected *p* < 0.05; Fig. 6). The results indicate that early-blind subjects have better speech recognition in noise and a greater ability to separate speech from noise.[Fig eN-CFN-0528-23F7]

**Figure 7. eN-CFN-0528-23F7:**
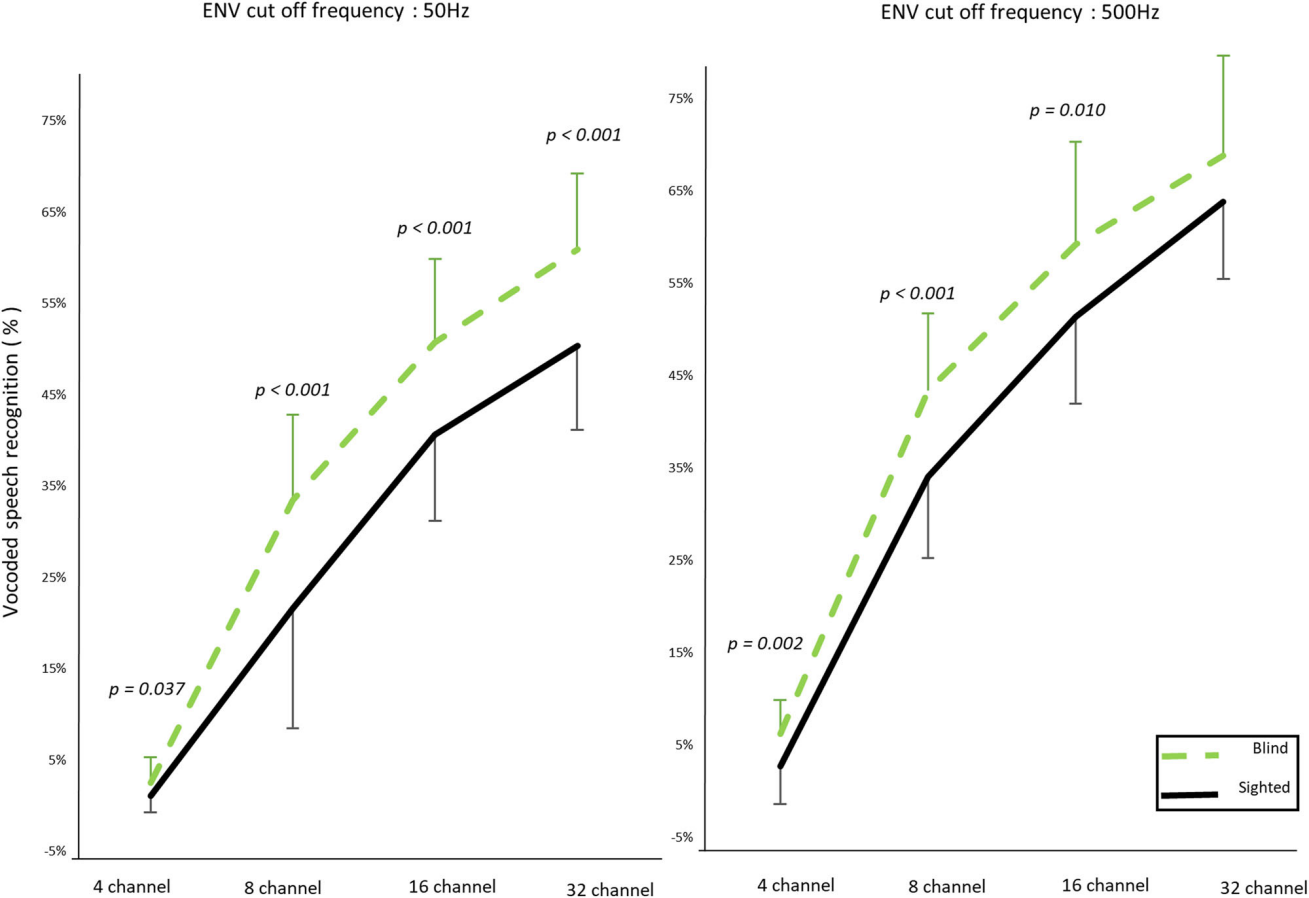
Vocoded speech recognition under envelope cutoff frequencies of 50 Hz (left panel) and 500 Hz (right panel). The early-blind group showed better recognition compared with the sighted group (*p* < 0.001). Group showed an interaction with channels (*p* = 0.007) but not with the envelope cutoff frequency (*p* = 0.057).

#### Vocoded speech recognition

Speech recognition was measured when both spectral and temporal information were degraded. The mixed three-way RM-ANOVA (two groups × four numbers of channels × two envelope cutoff frequencies) showed significant main effects of group (*F*_(1, 48)_ = 20.604; *p* < 0.001), number of channels (*F*_(3, 144)_ = 873.452; *p* < 0.001), and envelope cutoff frequency (*F*_(1, 48)_ = 256.051; *p* < 0.001). A significant three-way interaction was detected (*F*_(3, 144)_ = 2.628; *p* = 0.053). Group interacted with channels (*F*_(3, 144)_ = 4.184; *p* = 0.007) but not with the envelope cutoff frequency (*F*_(1, 48)_ = 3.815; *p* = 0.057; Table 3). In the post hoc tests, the early-blind subjects showed better noise-vocoded speech recognition than the sighted subjects across all channels with a 50 Hz envelope cutoff frequency (4 channels, *p* = 0.037; 8 channels, *p* < 0.001; 16 channels, *p* < 0.001; and 32 channels, *p* < 0.001; Bonferroni-corrected *p* < 0.05), except for the 32-channel test with a 500 Hz cutoff frequency (4 channels, *p* = 0.002; 8 channels, *p* < 0.001; 16 channels, *p* < 0.001; and 32 channels, *p* < 0.076; Bonferroni-corrected *p* < 0.05) (Fig. 7). The results indicate that early-blind subjects showed superior recognition compared with sighted subjects even under conditions of degraded auditory spectral and temporal resolution. Early-blind subjects demonstrated increased sensitivity to spectral degradation for speech recognition, as evidenced by the significant interaction between group and channel. However, there was no group difference in the impact of the temporal envelope.

**Table 3.** ANOVA table of vocoded speech recognition

	Sum of square	df	Mean square	*F*	*p*	$\eta_{G}^{2}$
Channels	11,986.85	3.00	3,995.62	873.45	<0.001	0.905
Envelop	453.690	1.000	453.690	256.051	<0.001	0.842
Group	345.960	1	345.960	20.604	<0.001	0.300
Channels*Group	57.420	3.00	19.140	4.184	0.007	0.080
Channels*Envelop	74.090	3	24.697	16.641	<0.001	0.257
Envelop*Group	6.760	1.000	6.760	3.815	0.057	0.074
En*Ch*Gr	11.700	3	3.900	2.6828	0.053	0.052
Residual (channels)	658.73	144.00	4.57			
Residual (envelop)	85.050	48.000	1.772			
Residual (channels*envelope)	213.710	144	1.484			

### EEG

It measures N2 and P3b. N2 reflects cortical responses related to the lexical selection process, involving cortical access to lexical information and semantic categorization (Finke et al., 2016). The P3b component is also associated with updating working memory, and prolonged latency may be interpreted as a slower stimulus evaluation (Beynon et al., 2005; Henkin et al., 2015).

**Figure 8.**
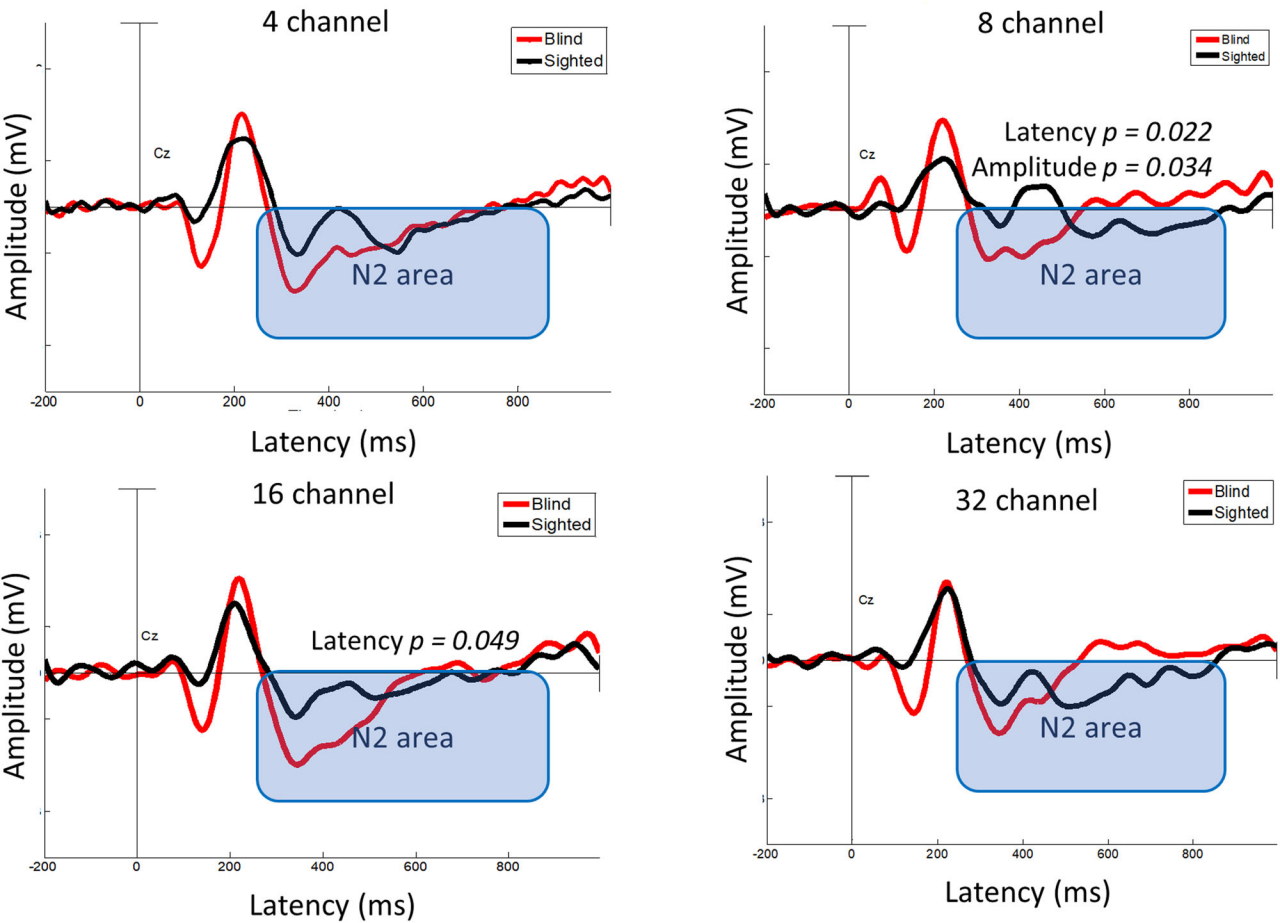
N2 latency and amplitude. The early-blind group showed a shorter latency in the 8-channel test (*p* = 0.022) and the 16-channel test (*p* = 0.049) and a greater amplitude in the 8-channel test (*p* = 0.034) compared with the sighted group (Bonferroni-corrected *p* < 0.05).

Mixed two-way RM-ANOVA (two groups × four numbers of channels) was conducted for both N2 latency and amplitude. The analysis revealed a significant effect of number of channels for latency (*F*_(3, 144)_ = 42.615; *p* < 0.001) and amplitude (*F*_(2.509, 120.423)_ = 5.353; *p* = 0.003). However, the group effect was not significant for either latency (*F*_(1, 48)_ = 2.475; *p* = 0.122) or amplitude (*F*_(1, 48)_ = 2.477; *p* = 0.122). In addition, the interaction between the number of channels and group was not significant for latency (*F*_(3, 144)_ = 2.561; *p* = 0.057) or amplitude (*F*_(2.509, 120.423)_ = 1.433; *p* = 0.240). Post hoc tests indicated that the early-blind group exhibited shorter latency than the sighted group for the 8-channel and 16-channel (8-channel, *p* = 0.022; 16-channel, *p* *=* 0.049; Bonferroni-corrected *p* < 0.05) tests, with a greater amplitude for the 8-channel test (*p* = 0.034; Bonferroni-corrected *p* < 0.05; Fig. 8).

**Figure 9.**
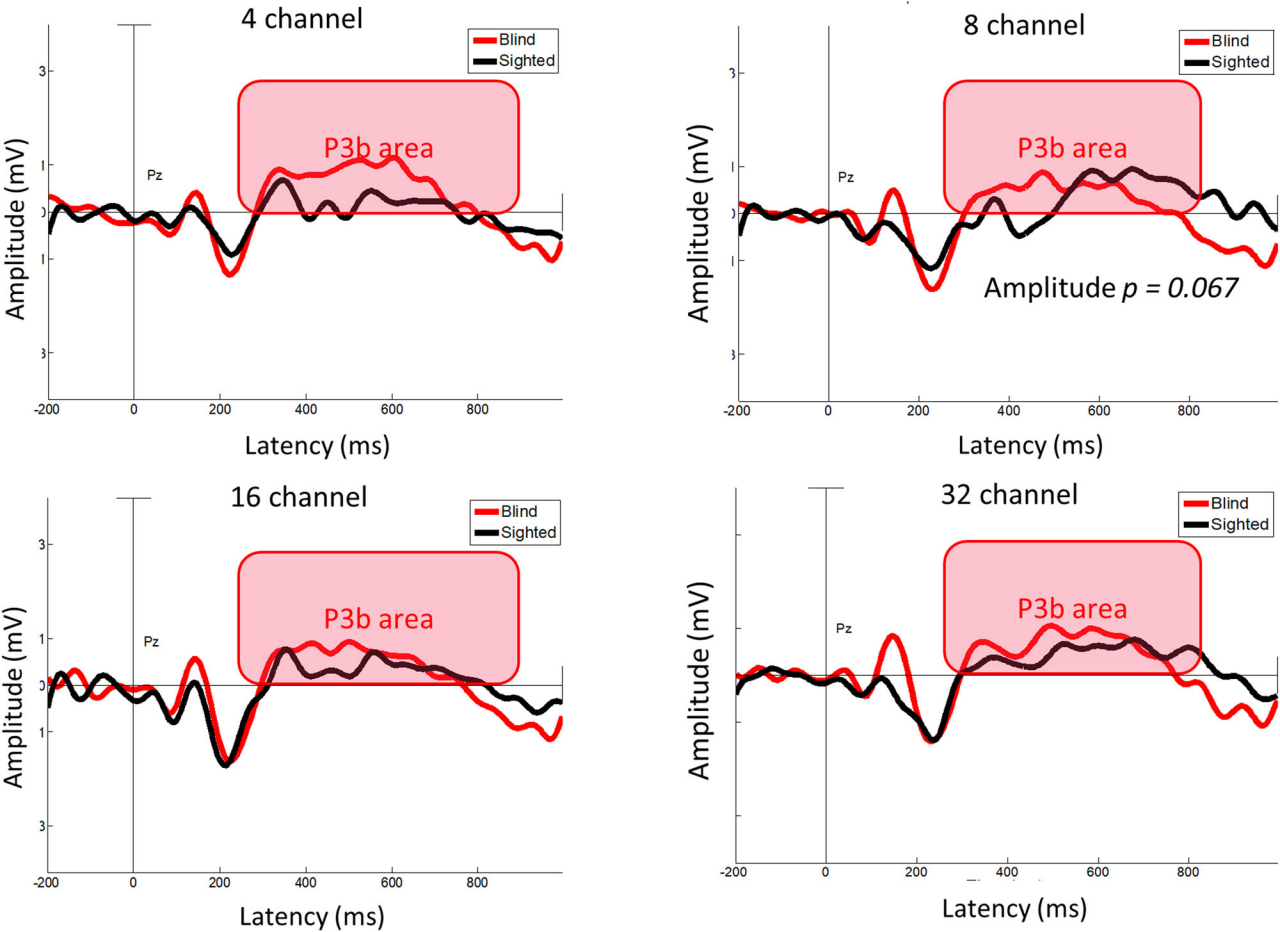
P3b latency and amplitude. The early-blind group tended to show a greater amplitude in the 8-channel test compared with the sighted group (*p* = 0.067; Bonferroni-corrected *p* < 0.05).

Similarly, the mixed two-way RM-ANOVA was conducted for P3b latency and amplitude (two groups × four numbers of channels) and revealed a significant effect of number of channels for both latency (*F*_(3, 144)_ = 8.739; *p* < 0.001) and amplitude (*F*_(3, 144)_ = 4.286; *p* = 0.006). However, the group effect was not significant for either latency (*F*_(1, 48)_ = 0.008; *p* = 0.927) or amplitude (*F*_(1, 48)_ = 1.906; *p* = 0.174). Furthermore, the interaction between the number of channels and group was not significant for latency (*F*_(3, 144)_ = 0.020; *p* = 0.996) or amplitude (*F*_(3, 144)_ = 1.352; *p* = 0.260). The post hoc tests for P3b confirmed a trend toward greater amplitude in the 8-channel test (*p* = 0.067; Bonferroni-corrected *p* < 0.05; Fig. 9).

## Discussion

In this study, early-blind subjects exhibited superior performance in both monosyllabic and sentence tasks compared with sighted subjects. Several studies have reported enhanced vowel perception (Ménard et al., 2009; Arnaud et al., 2018) and ultrafast speech comprehension (Dietrich et al., 2011; Hertrich et al., 2013) in early-blind individuals. However, other studies found no differences between early-blind and sighted individuals for two-syllable perception (Gougoux et al., 2009; Shim et al., 2019), monosyllable perception (Guerreiro et al., 2015; Bae et al., 2022), and sentence perception (Gordon-Salant and Friedman, 2011). The novel aspect of our study is that the word-in-noise test was performed at noise levels exceeding −4 dB SNR. Consistent with our hypothesis, the superior speech recognition of early-blind subjects was confirmed at high noise intensity. However, the expectation that “as the SNR increases, the speech recognition ability in early-blind subjects would show even greater superiority over sighted subjects” was not confirmed. Regarding the sentence test, both groups exhibited superior performance in ISTS over SSN, which could be attributed to masking release mechanisms (Christiansen and Dau, 2012; Biberger and Ewert, 2019). The significant interaction between group and noise type implies that blind subjects use masking release more efficiently than sighted subjects. The consistent advantage of blind subjects under both noise conditions during the sentence tests may be partially reliant on their superior working memory, as demonstrated by the digit span test. Numerous studies have highlighted that blind individuals excel in working memory tasks, including the digit span test (Rokem and Ahissar, 2009; Withagen et al., 2013) and the word memory test (Raz et al., 2007). Raz et al. (2007) postulated that early-blind individuals develop compensatory serial strategies due to the absence of visual input, heavily relying on spatial memory for perception. This heightened proficiency may arise from actual brain reorganization in blind individuals whose brains become more adapted to spatial, sequential, and verbal information (Cornoldi and Vecchi, 2000) as well as tactile stimuli (Rauschecker, 1995; Sterr et al., 1998; Bavelier and Neville, 2002).

Previous studies have indicated that blind individuals with normal hearing thresholds have superior auditory spectral resolution (Wan et al., 2010; Voss and Zatorre, 2012; Arnaud et al., 2018; Shim et al., 2019) and temporal resolution (Muchnik et al., 1991; Weaver and Stevens, 2006; Shim et al., 2019) compared with sighted individuals. However, considering blind individuals are dependent on their auditory performance but may develop age-related hearing loss or experience dual audiovisual impairments, the significance of their auditory performance becomes more pronounced. Yet, few studies have enrolled blind individuals with hearing impairments.

Auditory spectral resolution depends primarily on the active movement of outer hair cells, and initial cochlear damage starts from the outer hair cells; the disturbance of the active movement of the outer hair cells makes the basilar membrane response more linear and broadly tuned (Glasberg and Moore, 1986; Dubno and Schaefer, 1995; Oxenham and Bacon, 2003). The reduced compression and the broadening of the auditory filters negatively affect both frequency selectivity and temporal resolution (Glasberg and Moore, 1986; Moore et al., 1988; Moore and Oxenham, 1998; Oxenham and Bacon, 2003; Moon et al., 2015; Shim et al., 2019). Spectral and temporal degradation in sound affects the coding of sounds in both the peripheral and central auditory systems. When exposed to spectral degradation in sound, difficulty arises in frequency filtering, leading to the auditory nerves receiving incomplete sound information. Consequently, the brain may fail to recognize sounds properly (Edwards, 2003). Impaired temporal acuity hinders the encoding of amplitude modulation signals in the auditory nerve and brainstem which can be represented by a decline in phase-locking depending on the modulation frequency (Walton, 2010). Furthermore, there is difficulty in detecting or perceiving changes in speech because auditory neurons may become less responsive to rapid changes in sound.

In our study, even with spectral and temporal degradation, early-blind subjects showed better speech discrimination than sighted subjects. Nevertheless, speech recognition declined more as spectral degradation worsened, indicating a stronger influence on blind subjects in compromised spectral conditions than sighted subjects. The impact of the temporal envelope displayed no group difference, contrasting with the notable effect of spectral information levels. Prior research noted that the advantage of spectral resolution requires prolonged visual loss, with positive correlations between blindness duration and spectral resolution (Shim et al., 2019), and negative correlations with age at the blindness onset (Gougoux et al., 2004). However, there is no evidence supporting a correlation between blindness duration and temporal resolution (Shim et al., 2019). Auditory spectral resolution may take a long time for functional enhancement, while temporal resolution improves more rapidly after visual deprivation, possibly due to distinct plastic changes in the brain caused by long-term visual loss affecting both resolutions. This disparity may influence the impact of degraded spectral and temporal cues on speech recognition for each resolution.

In a recent study like ours, researchers used 8-channel and 1-channel noise–vocoded sentences with early-blind and sighted individuals, employing magnetoencephalography for measurement (Van Ackeren et al., 2018). The magnetoencephalography analysis revealed increased synchronization in the primary visual cortex among early-blind individuals, along with enhanced functional connectivity between temporal and occipital cortices. Despite these neural differences, behavioral tests assessing vocoded sentence comprehension showed no significant between-group variations. Our study diverges from Van Ackeren et al.'s findings, as our early-blind group outperformed in monosyllable and sentence recognition. While Van Ackeren et al. focused on sentence comprehension, our emphasis was on recognizing individual words within sentences.

It has been acknowledged that humans rely more on top–down processing when the spectral or temporal information in the speech signal is degraded (Shannon et al., 1995; Davis et al., 2005; Obleser and Eisner, 2009; Peelle and Davis, 2012). N2 and P3b responses can measure the top–down mechanisms involved in speech comprehension. The N2 component is sensitive to perceptual novelty associated with access to lexical information and semantic categorization (Schmitt et al., 2000; Van den Brink and Hagoort, 2004). Meanwhile, the P3b component is associated with updating working memory, and prolonged latencies may be interpreted as slower stimulus evaluation (Beynon et al., 2005; Henkin et al., 2015). The standards and targets usually differ by a simple physical feature; prior studies using P3b examined tone discrimination (Kalaiah and Shastri, 2016; Perez et al., 2017) and used complex words (Kotchoubey and Lang, 2001; Balkenhol et al., 2020). Finke et al. (2016) used an oddball paradigm that required individuals to semantically classify words as living or nonliving entities. These additional circuits include retrieving word meanings from our mental lexicon and the circuits involved in categorizing words based on these meanings, which are reflected by a delayed latency and a greater amplitude of the P3b component as a function of the intensity of background noise (Henkin et al., 2008; Finke et al., 2016; Balkenhol et al., 2020). We observed a distinct effect of the number of channel bands on speech intelligibility and the N2 and P3b responses. As the number of channel bands decreased, the N2 and P3b amplitudes decreased, and their latencies increased. Strauss et al. (2013) reported that the N400 responses showed a similar channel effect when using the classical congruent/incongruent semantic paradigms in sentences. Unlike the sentence paradigm, the use of monosyllable words allowed us to minimize the redundancy of cues, reduce top–down expectations in the context (Bae et al., 2022), and control for individual differences in education and attention ability (Roup et al., 2006; Kim et al., 2008). In this study, differences between the early-blind group and the sighted group were only evident in the 8- and 16-channel tests. The results of N2 and P3b responses in the current study partially suggest that better speech perception in early-blind subjects compared with that in sighted subjects, even in situations of spectral and temporal degradation, could be primarily attributed to differences in top–down semantic processing. The brains of blind individuals may react more rapidly and robustly to lexical selection and semantic categorization processes. Numerous neuroimaging studies have revealed the recruitment of the occipital cortex in humans by auditory signals to perform auditory functions in a compensatory cross-modal manner, which correlates with improved auditory performance (Leclerc et al., 2000, 2005; Weeks et al., 2000; Voss et al., 2008, 2014; Gougoux et al., 2009; Voss and Zatorre, 2012). Early-blind individuals, having thicker cortical layers compared with those with nonvisual impairments, exhibit superior performance in pitch and melody discrimination (Voss and Zatorre, 2012). Their thicker cortices might be due to what is known as “use-dependent plasticity” (Gougoux et al., 2004; Hamilton et al., 2004). Heightened pitch discrimination in blind individuals has been directly linked to the degree of structural neuroplasticity in the cortex (Voss and Zatorre, 2012; Voss et al., 2014).

Degradation affects speech intelligibility and is known to be reflected in EEG. Studies using spectrally degraded vocoded speech have shown that vocoded speech resulted in smaller evoked potentials such as N450 and N400 compared with clear speech, implicating less robust semantic integration in spectrally degraded speech (Van Wassenhove et al., 2005). The effect of temporal degradation has been frequently studied in language-impaired populations, where manipulating the duration of speech resulted in diminished amplitudes in components like P2 and N2/N4, suggestive of diminished function in content encoding in the language-impaired group (Ceponiene et al., 2009). In real-world communication, which is inherently multimodal for sighted and hearing individuals, multisensory integration is known to bring benefits such as increased accuracy, speed (Besle et al., 2004), and attention. It is widely agreed that visual speech speeds up cortical processing of auditory signals within 100 ms poststimulus onset, with N1 and P2, the most robust auditory event-related potentials, significantly reduced in amplitude by the influence of visual speech (Van Wassenhove et al., 2005). Early cochlear implant users showed comparable auditory and visual potentials to their normal hearing peers, and their auditory activation became stronger in the AV compared with AO mode, likely due to reinforcement after implantation (Alemi et al., 2023). Furthermore, the N1 and P2 components of auditory-evoked potentials are known to be suppressed due to AV interactions, resulting in earlier and smaller amplitudes compared with when no visual information is provided (Van Wassenhove et al., 2005). It is expected that blind individuals have advantages in speech recognition due to their high sensitivity to spectral information.

A United States study found that ∼21% of seniors face both visual and hearing impairments by 70 years of age, with the estimated 45,000–50,000 individuals in the United States living with both hearing and visual impairments (Brabyn et al., 2007). If early-blind individuals experience age-related hearing decline, their mobility challenges, such as discerning sound direction with a cane, may increase navigation hazards (Brabyn et al., 2007). Blind travelers heavily rely on subtle auditory cues for orientation, making it crucial to address combined impairments. However, research on combined visual and hearing impairments is rare. A recent multi-institutional study comparing cochlear implant outcomes in deaf–blind and deaf-only children showed no significant differences in Categories of Auditory Performance scores at 12 and 24 months postimplantation. However, deaf–blind children exhibited lower speech intelligibility rating scales and word recognition scores compared with deaf-only children (Daneshi et al., 2022).

The current study is the first to compare speech recognition and relevant cortical-evoked potentials between early-blind subjects and sighted subjects under conditions of degraded auditory spectral and temporal resolution. The results have implications for designing interventions and support systems for individuals with combined visual and hearing impairments. Understanding speech processing in blind individuals in the presence of spectral and temporal degradation can assist clinicians in developing more effective strategies to improve speech recognition for blind individuals with hearing loss.

One limitation of the study is that while spectral resolution was compared using four numbers of channels, the temporal envelope resolution was only compared using two cutoff frequencies. Therefore, it would be necessary to further investigate these conditions by finely adjusting the temporal envelope cues in future studies. Furthermore, this study did not target individuals with actual degraded spectral or temporal resolution; rather, we focused on young adults with normal hearing and used simulated vocoded speech. Therefore, this study recruited participants exclusively from their 20s and 30s. To investigate the auditory performance and central auditory processing in individuals with combined visual and hearing impairments, a study of elderly individuals with visual and hearing impairments is needed.

## Synthesis

Reviewing Editor: Frederike Beyer, Queen Mary University of London Decisions are customarily a result of the Reviewing Editor and the peer reviewers coming together and discussing their recommendations until a consensus is reached. When revisions are invited, a fact-based synthesis statement explaining their decision and outlining what is needed to prepare a revision will be listed below. The following reviewer(s) agreed to reveal their identity: Jinxing Wei. Note: If this manuscript was transferred from JNeurosci and a decision was made to accept the manuscript without peer review, a brief statement to this effect will instead be what is listed below.

As you can see, both reviewers highlight the interest of your work to the field, but raise several points that need more detailed explanation or discussion. Please see the below review reports for details.

Reviewer 1 comments:

The author provides a comprehensive overview of the research focus, hypotheses, and methodology regarding the comparison of speech recognition abilities and cortical auditory responses between early-blind and sighted individuals under conditions of degraded spectral and temporal resolution. The author found that, early blind subjects demonstrated speech recognition advantages, even in the presence of spectral and temporal degradation, over sighted subjects.

Major point:

Here are some suggestions for enhancing the clarity and coherence of the introduction part: The author should provide a brief background on the importance of understanding auditory perception and speech recognition abilities in blind individuals, especially under conditions of degraded auditory information.

The subjects in both groups are around 30 years old, while hearing decline in one's thirties is not commonly observed, can the author provide the more detail data about comparison between different age stage (over 40)? Or the author should discuss this in the article.

Mini point:

In figure2, what is the axis label? Please label it in the figure.

In figure 5-7, the SD bar is hard to discriminate, change the different color.

Reviewer 2 comments:

General comments:

• Since the focus of the study is on the effects of spectral and temporal degradation, the current manuscript lacks a discussion of how audiovisual speech processing may be impacted by degradation of spectral or temporal or a combination of both.

• For the rationale of this study, I believe a more compelling rationale would be the fact that speech recognition is even a more important task in blind individuals than in sighted ones because blinded individuals have to entirely rely on their hearing to understand speech without being able to incorporate visual cues through multimodal processes such as lipreading.

Introduction:

• Line 68-69: Please state your specific hypothesis or hypotheses.

• Lines 70-72: Why only "to simulate temporally degraded speech"? Processing speech with a noise-vocoder with varying number of channels also changes spectral information. Please clarify.

• Lines 68-83: Please discuss how spectral and temporal degradation may impact EEG components and how such effects are expected to differ between only auditory versus audio-visual speech processing.

Materials and Methods:

• Please specify the age at onset of blindness for the studied population.

• What was the rationale for choosing the temporal envelope cutoff frequencies of 50 Hz and 500 Hz?

• Line 107: Add proper citations for each behavioral test.

• Lines 123-124: I wonder why SNR of 0 dB SPL was not included in the test.

• Figure 1: Indicate "Noise-vocoded Speech Sound" for the output.

Results:

• For all the stats, please report the actual p-values.

• Lines 243-249: Was the normality of the data checked before running t test? Also, please make some brief statement on what these results indicate. Of course, you have discussed them in the discussion section, but a brief discussion (a sentence after reporting each result) here would help.

• Lines 257-262: The direction of the significant main effect of group (which group performed better) is not clear in these results. Please clarify.

• Overall, the results section seems shallow with some raw reports that are not well expanded.

Discussion:

• Although the results are interesting, there is a major lack in connecting the findings to how processing of spectral and temporal information may be different between two groups at the level of peripheral auditory processing and central auditory processing. Discussion needs to be significantly improved to precisely make these connections as related to these findings. For example, it is not clear how these results add to the current knowledge on how the neural coding of spectral and temporal information (e.g., pitch, formants, duration) differ between blinded and sighted individuals.

• The specific clinical implications of the findings are not elucidated clearly. It's important to address how these findings can inform clinicians in their clinical practice.

## Author Response

Synthesis Statement for Author (Required):

As you can see, both reviewers highlight the interest of your work to the field, but raise several points that need more detailed explanation or discussion. Please see the below review reports for details.

Reviewer 1 comments:

The author provides a comprehensive overview of the research focus, hypotheses, and methodology regarding the comparison of speech recognition abilities and cortical auditory responses between early-blind and sighted individuals under conditions of degraded spectral and temporal resolution. The author found that, early blind subjects demonstrated speech recognition advantages, even in the presence of spectral and temporal degradation, over sighted subjects.

Thank you for reviewing our manuscript and providing valuable feedback. Below, I have addressed your comments point by point and made every effort to thoroughly revise the main text accordingly.

Major point:

Here are some suggestions for enhancing the clarity and coherence of the introduction part: The author should provide a brief background on the importance of understanding auditory perception and speech recognition abilities in blind individuals, especially under conditions of degraded auditory information.

Based on the reviewer's feedback, I have revised the introduction to incorporate why auditory perception is crucial for blind individuals compared to sighted individuals, and I have added content regarding the impact of spectral and temporal degradation in sound on individuals with visual impairments. Additionally, I have clarified the hypotheses.

The subjects in both groups are around 30 years old, while hearing decline in one's thirties is not commonly observed, can the author provide the more detail data about comparison between different age stage (over 40)? Or the author should discuss this in the article.

In this study, there were 12 early-blind participants in their 20s and 13 in their 30s, along with 12 sighted subjects in their 20s and 13 in their 30s; however, there were no participants in their 40s. The exclusion of participants in their 40s was intentional due to the potential presence of mild hearing impairment in this age group. Therefore, it was discussed that further research involving elderly participants is needed (line 504-505).

Mini point:

In figure2, what is the axis label? Please label it in the figure.

The y-axis is presented in frequency (Hz) units, and the label has now been added to the figure.

In figure 5-7, the SD bar is hard to discriminate, change the different color.

We altered the SD representation for blind individuals to be depicted in green and for sighted individuals in black. However, because of significant overlap, we chose to display only positive values for visually impaired individuals and negative values for sighted individuals Reviewer 2 comments:

Thank you for reviewing our manuscript and providing valuable feedback. Below, I have addressed your comments point by point and made every effort to thoroughly revise the main text accordingly.

General comments: • Since the focus of the study is on the effects of spectral and temporal degradation, the current manuscript lacks a discussion of how audiovisual speech processing may be impacted by degradation of spectral or temporal or a combination of both.

Based on the reviewer's feedback, we have incorporated this content into the discussion section. Here is the revised discussion (line 380-395). • For the rationale of this study, I believe a more compelling rationale would be the fact that speech recognition is even a more important task in blind individuals than in sighted ones because blinded individuals have to entirely rely on their hearing to understand speech without being able to incorporate visual cues through multimodal processes such as lipreading.

Based on the reviewer's feedback, we have incorporated the compelling rationale along with the authors' previous research findings into the introduction (line 51-60).

Introduction: • Line 68-69: Please state your specific hypothesis or hypotheses.

We have comprehensively revised the introduction and clearly articulated the authors' specific hypotheses based on the reviewer's feedback (line 67-69). • Lines 70-72: Why only "to simulate temporally degraded speech"? Processing speech with a noise-vocoder with varying number of channels also changes spectral information. Please clarify.

In this study, spectral degradation was achieved by using 4, 8, 16, and 32 channels to visualize spectral information, while temporal degradation was achieved by setting the cutoff frequency to 50 and 500. To prevent confusion, the corresponding sentence has been modified to clarify its meaning (line 72-74). • Lines 68-83: Please discuss how spectral and temporal degradation may impact EEG components and how such effects are expected to differ between only auditory versus audio-visual speech processing.

Due to the character limit in the introduction, we briefly mentioned these points in the introduction (line 87-94) and provided more detailed discussions on the results of other studies in the discussion section (line 460-479).

Materials and Methods: • Please specify the age at onset of blindness for the studied population.

Some blind subjects were diagnosed with congenital blindness, but some couldn't recall the exact onset of blindness. In this study, only participants who had received a diagnosis of blindness before the age of one and had no recollection of vision were included. Hence, the term 'early-blind' was used instead of 'congenital-blind'. Due to these reasons, it's difficult to pinpoint the exact onset of blindness for each subject. • What was the rationale for choosing the temporal envelope cutoff frequencies of 50 Hz and 500 Hz? The cutoff frequency of the low-pass filter for temporal envelope extraction was applied at both 50 Hz and 500 Hz, depending on whether fundamental frequency (F0)-related periodicity cues were included (i.e., absence of F0 cue at 50 Hz versus presence of F0 cue at 500 Hz cutoff frequency) (line 182-185).

We referenced the method used by Ananthakrishnan et al., 2017, and confirmed that the fundamental frequency of the monosyllabic words used in our experiment ranged from 50 to 500 Hz. • Line 107: Add proper citations for each behavioral test.

We added (line 116-118). • Lines 123-124: I wonder why SNR of 0 dB SPL was not included in the test.

In our previous study (Bae et al.,2022), we compared the speech perception of early-blind subjects and sighted subjects across five different SNRs: -18, -16, -12, -8, and -4, using the same monosyllable set as in the current research. Monosyllable perception in noise tended to be better in early-blind subjects than in sighted subjects at a SNR of -8 (p = 0.054) however, the results at SNR -4, 0, +4, and +8 did not differ. Therefore, in this study, we designed conditions with low SNRs (signal-to-noise ratios; high noise levels) (line 134-140). • Figure 1: Indicate "Noise-vocoded Speech Sound" for the output.

Correction complete Results: • For all the stats, please report the actual p-values.

Correction complete • Lines 243-249: Was the normality of the data checked before running t test? We reanalyzed the results of the digit span test using the Mann-Whitney test after confirming that some data did not follow a normal distribution based on the Kolmogorov-Smirnov test. We acknowledge that the oversight of not thoroughly confirming normal distribution was a mistake on the part of the authors. However, fortunately, the statistical results showed little to no difference.

We have updated the Statistical Analysis section (line 260-262) and the section reporting the results of the Digit Span Test (line 277 and 280) to reflect the inclusion of the Mann-Whitney test.

Also, please make some brief statement on what these results indicate. Of course, you have discussed them in the discussion section, but a brief discussion (a sentence after reporting each result) here would help.

Following the reviewer's feedback, we have added brief interpretations of the implications of each results section. • Lines 257-262: The direction of the significant main effect of group (which group performed better) is not clear in these results. Please clarify.

The direction of the significant main effect of group has been indicated (line 287 and line 294). • Overall, the results section seems shallow with some raw reports that are not well expanded.

We have updated the Results section to improve understanding.

Discussion: • Although the results are interesting, there is a major lack in connecting the findings to how processing of spectral and temporal information may be different between two groups at the level of peripheral auditory processing and central auditory processing. Discussion needs to be significantly improved to precisely make these connections as related to these findings. For example, it is not clear how these results add to the current knowledge on how the neural coding of spectral and temporal information (e.g., pitch, formants, duration) differ between blinded and sighted individuals.

We have added content suggesting that the differences in the processing of degraded spectral and temporal sound between the two groups are primarily attributed to differences in top-down semantic processing (line 420-423 and line 445-459).

In the Discussion section, we have incorporated neuroimaging study findings that can elucidate the mechanisms underlying the superior auditory performance observed in blind subjects (line 449-459). • The specific clinical implications of the findings are not elucidated clearly. It's important to address how these findings can inform clinicians in their clinical practice.

We have incorporated this information into the Discussion section (line 494-498).
