# Erratum: Choi et al., “The Impact of Spectral and Temporal Degradation on Vocoded Speech Recognition in Early-Blind Individuals”

**DOI:** 10.1523/ENEURO.0295-24.2024

**Published:** 2024-07-11

In the article, “The Impact of Spectral and Temporal Degradation on Vocoded Speech Recognition in Early-Blind Individuals,” by Hyo Jung Choi, Jeong-Sug Kyong, Jae Hee Lee, Seung Ho Han, and Hyun Joon Shim which published online on May 29, 2024, Hyo Jung Choi and Jeong-Sug Kyong should be listed as co-first authors due to their equal contributions to the work. The online version has been corrected to include the following statement as a footnote: “H.J.C. and J.-S.K. equally contributed as the co-first authors.”

